# Case Report: Low Dose of Valsartan/Sacubitril Leads to Successful Reversal of Acute Heart Failure in Chemotherapy-Induced Cardiomyopathy

**DOI:** 10.3389/fped.2021.639551

**Published:** 2021-02-25

**Authors:** Shih-Hsing Lo, Yi-Ching Liu, Zen-Kong Dai, I-Chen Chen, Yen-Hsien Wu, Jong-Hau Hsu

**Affiliations:** ^1^Department of Pediatrics, Kaohsiung Medical University Hospital, Kaohsiung, Taiwan; ^2^Department of Pediatrics, School of Medicine, College of Medicine, Kaohsiung Medical University, Kaohsiung, Taiwan

**Keywords:** pediatric heart failure, valsartan/sacubitril, cardiomyopathy, treatment, chemotherapy

## Abstract

Valsartan/sacubitril is a new agent approved for the treatment of chronic heart failure in adults, with a combination of angiotensin receptor inhibitor and neprilysin inhibitor. However, the benefit of valsartan/sacubitril in pediatric patients is unknown. We herein report its clinical benefit in a case of acute decompensated heart failure in chemotherapy-induced cardiomyopathy. This case suggests that in children with acute heart failure refractory to conventional medications, low dose of sacubitril/valsartan may be an effective therapy.

## Introduction

The importance of pediatric heart failure (PHF) has been emerging in clinical practice, with an incidence of 0.97–7.4 per 100,000 (1). Despite being a relatively uncommon condition, PHF is still an important cause of mortality and morbidity in the pediatric population. To date, there have been well-established guidelines for adult heart failure (HF) management. Indeed, since 2016, a novel agent with combination of angiotensin receptor and neprilysin inhibitor (ARNI), valsartan/sacubitril, had been introduced into both the European Society of Cardiology (ESC) and American College of Cardiology (ACC)/American Heart Association (AHA) guidelines for the management of adult HF (2, 3). However, the role and safety of this novel medication in children is not yet well-elucidated. Here we present a pediatric case of chemotherapy-induced dilated cardiomyopathy (DCM) with chronic HF with subsequent acute cardiac decompensation, which was successfully reversed by low dose of ARNI.

## Case Description

Acute decompensated HF was presented in a 7-year-old girl. Tracing back her past history, she had acute myeloid leukemia (AML) diagnosed at the age of 1 year, receiving chemotherapy according to Taiwan Pediatric Oncology Group-Acute Myeloid Leukemia (TPOG-AML) 2008 protocol, consisting of idarubicin, cytarabine, mitoxantrone, cyclophosphamide, etoposide, methotrexate, and mercaptopurine. However, due to AML relapse, part of the TPOG-AML 2008 protocol was repeated, with cumulative dose of 189 mg/m^2^ for idarubicin and 162 mg/m^2^ for mitoxantrone.

Cyclophosphamide dose was 200 mg/m^2^/day for 3–5 days within a course, with total used dose of 4 g/m^2^. For still poor-controlled disease, she then received peripheral blood stem cell transplantation (PBSCT) when she was 5 years old. The conditioning regimen prior to transplantation included busulfan (3.2 mg/kg), anti-thymocyte globulin (6 mg/kg), and high-dose cyclophosphamide (120 mg/kg). The heart function before transplantation was normal with left ventricular ejection fraction (LVEF) 58%, no cardiomegaly in chest radiography, with a serum level of B-type natriuretic peptide (BNP) of 78 pg/ml (normal <100). However, acute HF and DCM with reduced LVEF of 35% occurred 2 weeks after PBSCT, potentially induced by anthracyclines and cyclophosphamide. Her DCM was well-controlled by captopril (1 mg/kg/day) with stable LVEF of 50% and with Modified Ross/New York Heart Association (NYHA) functional class I for about 2 years.

However, 2 years after the PBSCT, acute HF developed with manifestations of pitting edema in the lower legs and shortness of breath at rest, compatible with Modified Ross/NYHA functional class IV. The echocardiogram during admission showed reduced LVEF of 22%. Despite fluid restriction and anti-congestive agents including furosemide, ramipril, bisoprolol, and spironolactone for 2 weeks, her clinical condition slightly improved to modified Ross/NYHA III and was discharged. However, the cardiac magnetic resonance imaging (MRI) demonstrated low LVEF of 18.3%, and cardiac catheterization showed cardiac index of 1.96 L/min/m^2^. Therefore, she was listed in the waiting list for heart transplantation and was followed regularly in our clinic.

**Figure 1:**
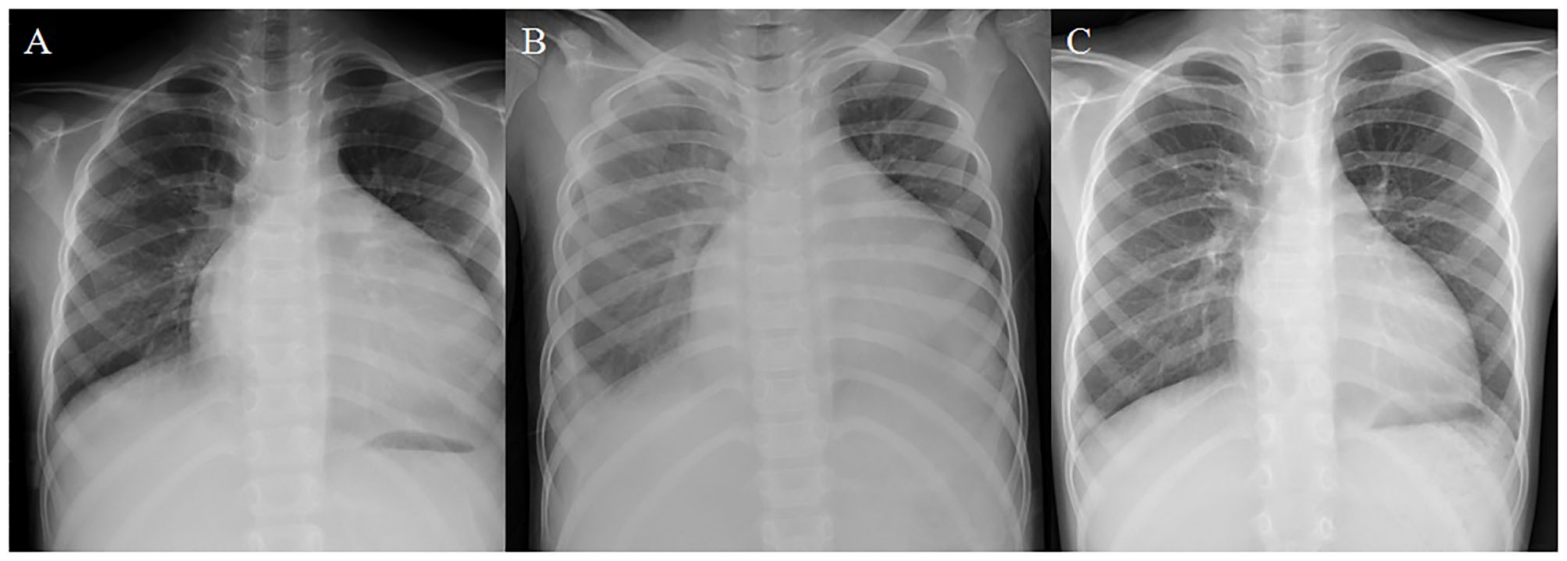
Remarkable improvement in chest X-ray was noted after the initiation of valsartan/sacubitril. **(A)** Chest X-ray upon admission on the second episode of acute decompensation. The cardiothoracic ratio (CTR) was 0.67. **(B)** Chest X-ray 2 weeks after aggressive treatment with conventional anti-congestive medications and fluid restriction. Cardiomegaly persisted with progressive right pleural effusion. **(C)** Chest X-ray 5 weeks after valsartan/sacubitril treatment. The CTR reduced to 0.53, and the pleural effusion had been resolved.

**Figure 2:**
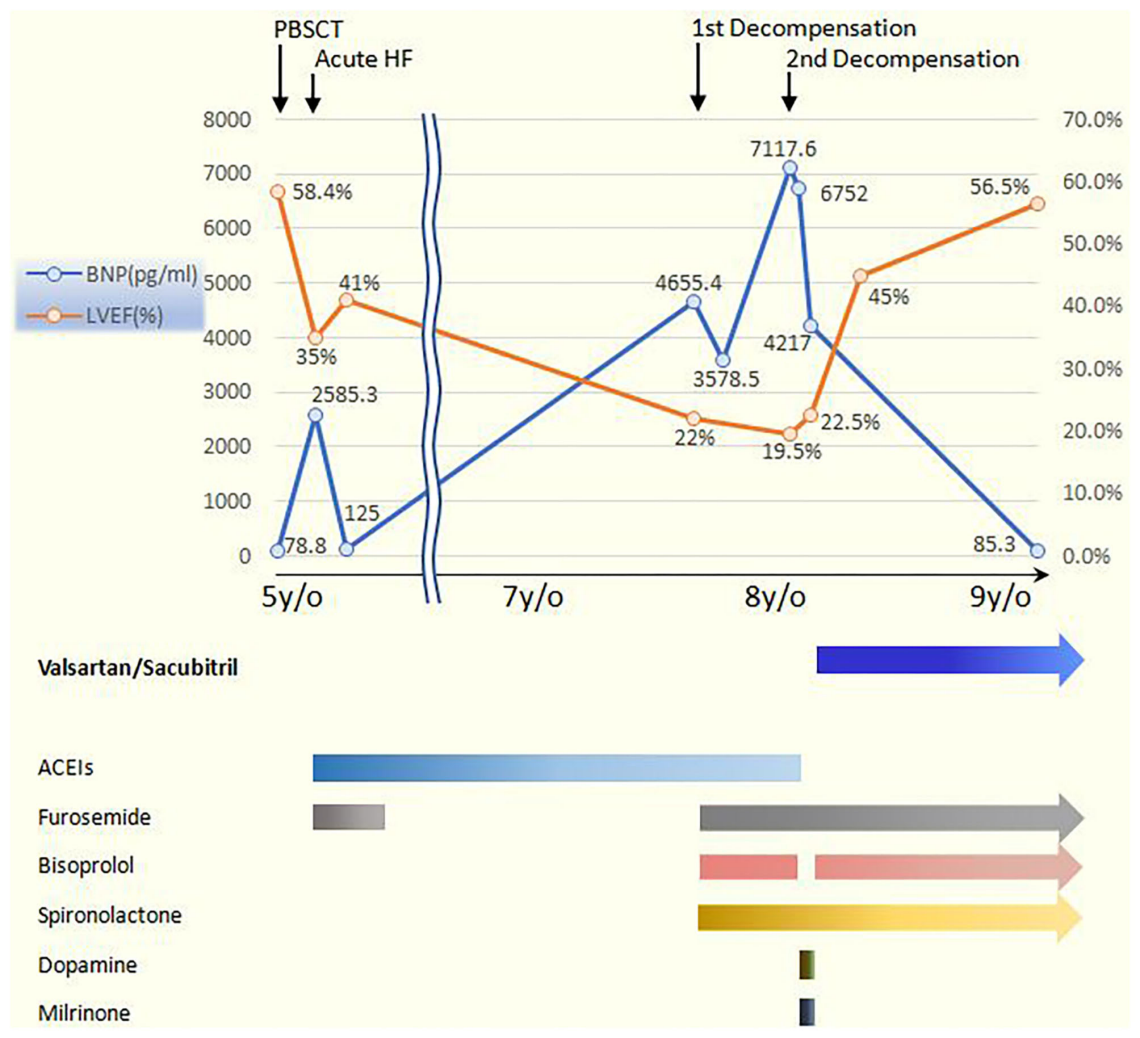
The serial changes of BNP and LVEF with medications adjustment in our patient. The baseline LVEF and BNP level before PBSCT when she was 5 years old were both normal. Acute HF occurred 2 weeks after PBSCT, and long-term ACEIs including captopril and ramipril were prescribed. Two episodes of acute decompensation later developed when she was 7 years old, while the BNP and LVEF both worsened. However, significant improvement occurred after the initiation of valsartan/sacubitril and persisted even at 1-year follow-up. BNP, B-type natriuretic peptide; LVEF, left ventricular ejection fraction; PBSCT, peripheral blood stem cell transplantation; HF, heart failure; ACEIs, angiotensin-converting-enzyme inhibitors.

Unfortunately, she was admitted again to our pediatric intensive care unit (PICU) 6 months later with another episode of acute cardiac decompensation, with Modified Ross/NYHA IV. The echocardiogram showed low LVEF of 19.5%. Despite meticulous management for 2 weeks, with fluid restriction, intravenous furosemide 2 mg/kg/day, oral spironolactone 2.25 mg/kg/day, ramipril 2.5 mg/m^2^/day, bisoprolol 1.25 mg/day, followed by intravenous infusion of inotropic agents with dopamine and milrinone, her HF progressed with cardiomegaly, and pleural effusion even further developed (Figures 1A,B). In the context of poor response to conventional medications, we changed ramipril to the ARNI (valsartan/sacubitril). We started with the dosage of 0.8 mg/kg/dose twice daily, without further adjustment because hypotension developed while increasing the dose. Fortunately, after the initiation of valsartan/sacubitril, her urine output doubled within 2 days, BNP rapidly declined within 5 days, and pleural effusion subsided after 10 days. Under unchanged dosage of valsartan/sacubitril, accompanied with furosemide, spironolactone, and bisoprolol, she was discharged out of PICU 14 days later and discharged 17 days later. The cardiothoracic ratio on chest X-ray decreased (Figure 1C), and LVEF improved (19.5% to 35.4%) 5 weeks later. Her LVEF shown by echocardiogram increased to 56.5% 1 year later (Figure 2), and her clinical status also significantly improved to Modified Ross/NYHA I. No further adverse effects such as hypotension, electrolyte imbalance, or impaired renal function were noted.

## Discussion

Pediatric heart failure is a complex clinical syndrome resulting from mostly congenital heart disease (CHD) and cardiomyopathies (CMs). In contrast, adult HF results from ischemic heart disease in most cases. Large randomized prospective trials on PHF are lacking, and so far no strong and evidence-based recommendations within well-established guidelines for the management of PHF had been proposed. On the other hand, the mechanism and pathogenesis of chemotherapy-related cardiomyopathies, like in our case, remain controversial and poorly understood. However, the treatment of HF in these CMs patients was similar to the general HF group.

In adults, valsartan/sacubitril is the first-in-class ARNI to treat chronic HF with reduced ejection fraction (HFrEF), but its benefit is so far not yet well-elucidated in pediatric populations (4). Our case suggests that in children with acute HF refractory to conventional medications, low dose of sacubitril/valsartan may be an effective therapy.

Valsartan/sacubitril is designed based on two mechanisms: (1) the blockade of renin-angiotensin-aldosterone system (RAAS) by valsartan to prevent the harmful profibrotic effect on cardiomyocyte and (2) the inhibition of neprilysin by sacubitril, an enzyme responsible for the breakdown of natriuretic peptides, with the combined effects of vasodilation, natriuresis, diuresis, and therefore reduction in both the pre-load and afterload (5–7). According to the 2017 ACC/AHA/HFSA guideline, valsartan/sacubitril has been listed as Class I B recommendation for adult patients with chronic HFrEF and as a replacement for angiotensin-converting-enzyme inhibitors (ACEI) and angiotensin II receptor blocker (ARB) for chronic symptomatic HFrEF (3). Similar suggestions were proclaimed in the 2016 ESC guideline (2). However, in pediatric populations its safety and equivalent benefits have not yet been completely understood.

Currently, there is an ongoing pediatric multicenter trial—PANORAMA-HF study (NCT00382525)—which will compare valsartan/sacubitril and enalapril in the treatment of pediatric HFrEF (8). Fortunately, positive mid-term results had prompted the recent approval from the American Food and Drug Association (FDA) in symptomatic pediatric HFrEF patients aged 1 year and older (9).

In our case, there were two inspiring implications regarding the use of valsartan/sacubitril in pediatric patients. First, even though FDA suggested initial dose in pediatric patients of 1.6 mg/kg twice daily, the prescribed dosage in our patient was only 0.8 mg/kg twice daily to avoid hypotension throughout the 1-year follow-up but still resulted in a remarkable improvement in her cardiac function. This may imply that a lower dose is effective enough to treat PHF with the benefit of less adverse effects, especially hypotension. In line with our observation in this case, similar results of improving cardiac function in relatively low doses of valsartan/sacubitril were shown in recent adult studies (10, 11). Second, the use of valsartan/sacubitril was generally recommended in adult patients with chronic HF NYHA II-III. In our case, we found that the acute decompensation status with NYHA IV could also be stabilized after the initiation of valsartan/sacubitril. These findings suggest that there can be some differences in the dose and indications between children and adults in this agent. Further studies are needed to substantiate our findings.

To date, there are still few case reports describing the use of valsartan/sacubitril in PHF. This case report suggests that it can be effective even in lower dose and acute decompensation status in children with HF. Further ongoing clinical trials of this novel medication may be needed to investigate the optimal dose and indications in pediatric populations.

## Data Availability Statement

The original contributions generated for the study are included in the article/supplementary material, further inquiries can be directed to the corresponding authors.

## Ethics Statement

Informed consent was obtained from the parents for publication of this case report.

## Author Contributions

J-HH carried out the studies. Y-CL, Z-KD, and I-CC participated in collecting data. S-HL drafted the manuscript. Y-HW helped to draft the manuscript. All authors contributed to the article and approved the submitted version.

## Conflict of Interest

The authors declare that the research was conducted in the absence of any commercial or financial relationships that could be construed as a potential conflict of interest.
